# Bioactivity-Guided Separation of Anti-Cholinesterase Alkaloids from *Uncaria rhynchophlly* (Miq.) Miq. Ex Havil Based on HSCCC Coupled with Molecular Docking

**DOI:** 10.3390/molecules27062013

**Published:** 2022-03-21

**Authors:** Pengfei Yu, Zhenshan Chen, Yuecheng Liu, Zhengwei Gu, Xiaoming Wang, Yaowen Zhang, Yanni Ma, Meiyue Dong, Zhenhua Tian

**Affiliations:** 1 School of Pharmacy, Shandong University of Traditional Chinese Medicine, Jinan 250355, China; 2019111084@sdutcm.edu.cn (P.Y.); 17862972335@163.com (Z.C.); z17860554048@163.com (Y.Z.); dmy1230@163.com (M.D.); 2 Inner MenGolia Mengqi Pharmaceutical Co. Ltd., Huhhot 011700, China; 3 Shandong Academy of Traditional Chinese Medicine, Jinan 250014, China; lyca3102021@163.com; 4 Experimental Center, Shandong University of Traditional Chinese Medicine, Jinan 250355, China; zhengweigudada@126.com (Z.G.); lmlwxm123@163.com (X.W.); lxyaner@163.com (Y.M.)

**Keywords:** *Uncaria rhynchophlly* (Miq.) Miq. ex Havil, HSCCC, alkaloids, cholinesterase inhibitory activity

## Abstract

As an important source of cholinesterase inhibitors, alkaloids in natural products have high potential value in terms of exerting pharmacological activities. In this study, a strategy for targeted preparation of cholinesterase inhibitors in *Uncaria rhynchophlly* (Miq.) Miq. ex Havil (UR) by high-speed counter-current chromatography was provided. In the method, a two-phase polar solvent system composed of ethyl acetate/*n*-butanol/water (1:4:5, *v*/*v*/*v*) was used, which isolated five alkaloids from the UR extract for the first time. All alkaloids were identified by HR-ESI-MS and NMR as 7-*epi*-javaniside (**1**), vincosamide (**2**), strictosamide (**3**), cadambine (**4**), and 3α-dihydrocadambine (**5**). The poorly resolved compounds **2** and **3** were separated by preparative HPLC (prep-HPLC). Among them, compounds **1**, **4**, and **5** were firstly obtained from UR. The purity of these plant isolates was 98.8%, 98.7%, 99.2%, 95.7%, and 98.5%, respectively. Compounds **1**–**5** exhibited an inhibitory effect on acetyl-cholinesterase and butyryl-cholinesterase with an IC_50_ from 1.47 to 23.24 µg/mL and 1.01 to 18.24 µg/mL. Molecular docking and inhibitory activities indicated that compound **1** showed stronger inhibitory activity on acetyl-cholinesterase and butyryl-cholinesterase.

## 1. Introduction

Cholinesterase inhibitors are a research hotspot in the treatment of Alzheimer’s disease (AD), neurological, hypertension, and other diseases. According to their resources, they can be divided into two categories: chemical synthesis and natural products. As an important source of natural cholinesterase inhibitors, alkaloids have great potential in the development of therapeutic drugs for AD. According to research, total alkaloids of *Uncaria rhynchophlly* (Miq.) Miq. ex Havil (UR) can significantly reduce the activity of cholinesterase in the serum of Alzheimer’s disease model rats [1]. The alkaloid components in UR as a cholinesterase inhibitor have still not been identified. In view of these benefits, a study on the separation and purification of alkaloids from UR is necessary.

UR is an evergreen vine of the Rubiaceae *Uncaria* and is an important traditional Chinese medicine, also called double-claw, upside-down thorn, eagle claw thorn, etc. Many phytochemicals, including alkaloids, triterpenes, phenol acids, and flavonoids are present in UR. The major active components are alkaloids of UR, which have anti-hypertensive [2], anti-convulsant [3], anti-epileptic [4,5], anti-inflammatory [6,7], and anti-cancer [8,9] activities. Traditional methods used to isolate complex mixtures from UR include silica gel chromatography, polyamide column chromatography, ODS column chromatography, high-pressure preparation of liquid phase, and others [10,11,12,13]. However, most of these methods have disadvantages such as adsorption, small sample loading, difficulty in separating isomers, and long preparation time. Therefore, traditional methods used for the separation of single compounds with low content, complex components, high polarity, unstable property, and similar structures are inefficient. These reasons lead to few reports in this direction. Therefore, a method for separating and purifying alkaloids is in urgent need.

High-speed counter-current chromatography (HSCCC) is a simple, continuous, and efficient liquid–liquid partition chromatography technology developed by Ito in the 1980s [14]. It does not use solid carriers and avoid irreversible adsorption and denaturation of samples. This processes the advantages of a high recovery rate and large preparation amount. CCC uses a biphasic liquid system: one phase is the stationary phase, the other is the mobile phase. Centrifugal fields are used to maintain the stationary phase stable and staying inside the CCC column. Therefore, HSCCC is widely used in the isolation and purification of natural products [15,16]. UR is rich in alkaloids and can be used as one of the natural sources of cholinesterase inhibitors. In view of the disadvantages of traditional separation methods such as slow separation speed, low yield, long time consumption, and the use of many organic reagents, HSCCC can make up for these disadvantages. Therefore, using HSCCC to separate alkaloids is an efficient and feasible method for the separation of cholinesterase inhibitors.

**Figure 1:**
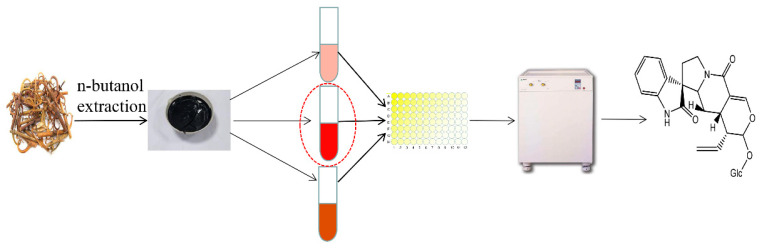
The effective method for targeted isolation of alkaloids with cholinesterase inhibitory activity from UR using HSCCC.

**Figure 2:**
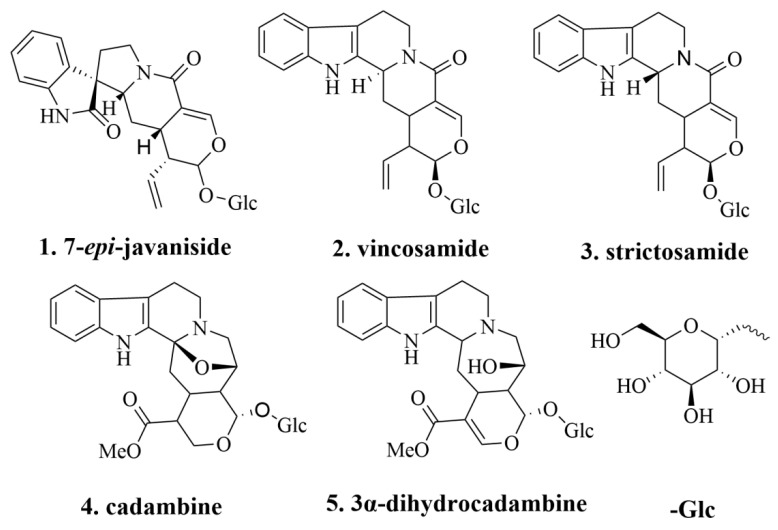
The structures of compounds **1**–**5**.

In this study, an effective method for targeted isolation of alkaloids with cholinesterase inhibitory activity from UR using HSCCC was established (Figure 1). The crude extract of *n*-butanol obtained from UR was segmented by macroporous adsorbent resin column chromatography (AB-8), the activity was determined, and the activity fraction was selected for HSCCC separation. Subsequent activity screening was performed on the isolated compounds to realize an activity-guided separation. Finally, a new method was successfully developed to isolate the five alkaloids rapidly and efficiently. The structures of the compounds were elucidated by HR-ESI-MS combined with ^1^H and ^13^C-NMR. The chemical structures of the isolated alkaloid are shown in Figure 2.

## 2. Results and Discussion

### 2.1. Selection of the HSCCC Solvent Systems

HSCCC is different from other traditional chromatographic because its stationary phase and mobile phase are both liquid. As a separation technology, HSCCC has advantages: (1) no solid carrier, no irreversible adsorption of samples, high sample recovery; (2) simple separation operation, short separation period, and less organic solvent consumption; (3) a wide range of solvent systems covering various polarities. Complex compounds are very suitable for the separation of complex components of plants; (4) the separation mode is diverse, and it has good adaptability to compounds with large differences in polarity; (5) the injection volume is large, which can be industrialized and meet the needs of clinical research.

**Figure 3:**
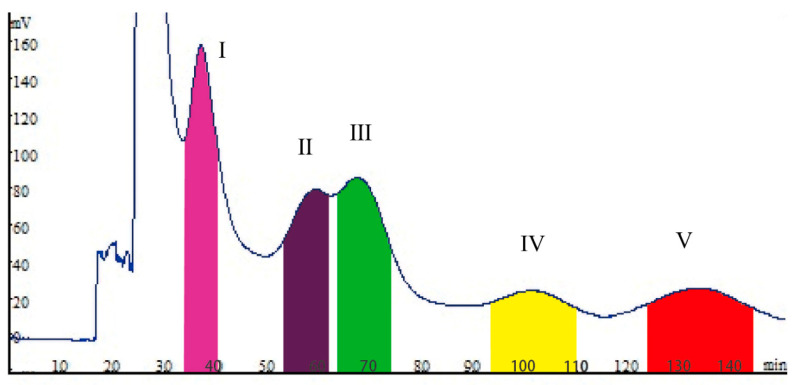
The HSCCC separation chromatogram of fractions I–V. HSCCC conditions: solvent system: ethyl acetate:*n*-butanol:water (1:4:5, *v*/*v*/*v*); stationary phase: UP; mobile phase: LP; rotating speed: 800 rpm; flow rate: 2.0 mL/min; separation temperature: 25 °C; detection wavelength: 220 nm; sample solution: extract 80 mg + UP 10 mL + MP 10 mL.

For HSCCC separation, it is crucial to choose a solvent system with moderate *K* values for the target compounds. The *K* value is the ratio of the mass of the target compound in the stationary phase to the mass in the mobile phase. Higher *K* values may result in overly large peak breadth and extended elution time. However, lower *K* values may result in poor peak resolution. In this study, an equal volume of the solvent system was taken from the upper and lower phases, then a small amount of UR crude extract was added, and the sample was fully dissolved in the solvent system by sufficient shaking. After the equilibrium was attained, the two phases were separated, dried under reduced pressure, and dissolved in methanol for analysis by HPLC. The partition value is the ratio of the concentration of the target compound in the stationary phase and mobile phase of the solvent system, which is very important for a suitable HSCCC separation. When the partition value of the target compound is within an appropriate range (0.5–2), HSCCC can obtain the target compound with high purity within the effective time. When the partition value of the target compounds is relatively small, it will lead to shorter retention time, faster peak emergence, low purity, and separation efficiency of the compound. When the partition value of compounds is too large, a large number of target compounds remain in the stationary phase, resulting in a very long separation time, which is not conducive to efficient separation. The partition ratio of each compound in the solvent system was calculated by the formula. In this study, several solvent systems with different proportions were tested. The results are described in Table 1. When the solvent systems composed of methyl tert-butyl ether:acetonitrile:water (4:1:5, *v*/*v*/*v*) were used, the *K* values were large, especially for compounds **3** and **4**. Thus, it became difficult to elute target compounds. Then, the solvent system composed of *n*-butanol:water (1:1, *v*/*v*) was tested but was not suitable due to a lower *K* value. Therefore, it was once again difficult to separate target compounds. Moreover, the solvent systems composed of ethyl acetate:*n*-butanol:water with different proportions were tested. When the ratio (4:1:5, *v*/*v*/*v*) was used, the *K* value of compound **1** was relatively low, which may lead to a short retention time and low purity. The *K* values of other compounds indicated that the compounds were mainly distributed in the lower phase, and their distribution in the upper phase can be increased by adjusting the upper phase solvent system. This showed that the chemical composition was mainly concentrated in the lower phase, which can be improved by increasing the proportion of the upper phase solvent. Then, the ratios of 4:0.5:5, 3:2:5, 1:4:5, and 1:5:5 were tested. The test results showed that when the proportion of ethyl acetate decreases, the proportion of *n*-butanol increases will increase the *K* value of the five compounds. Therefore, the two-phase solvent system consisting of ethyl acetate:*n*-butanol:water (1:4:5, *v*/*v*/*v*) was selected as the optimal solvent system for the experiment. The separation results are shown in Figure 3.

### 2.2. HSCCC Process and HPLC Analysis

**Figure 4:**
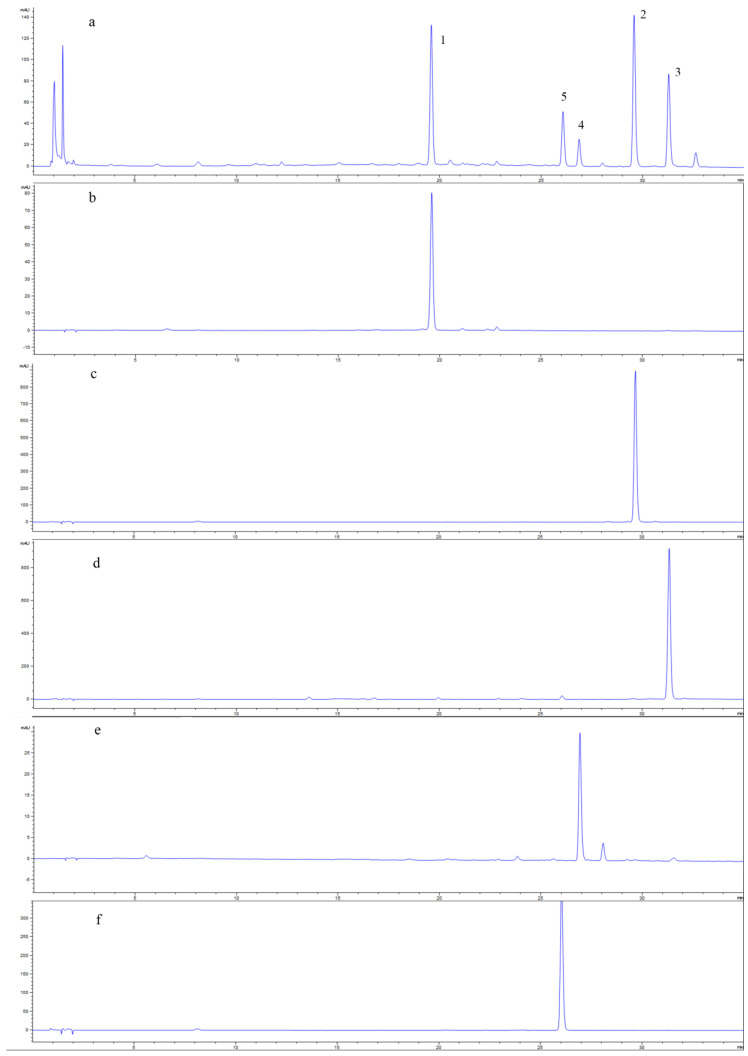
HPLC analysis compounds ((**a**), extract crude; (**b**–**f**), compound **1**–**5**).

The HSCCC process was carried out using ethyl acetate:*n*-butanol:water (1:4:5, *v*/*v*/*v*) with other parameters: revolution speed, 800 rpm; flow rate, 2.0 mL/min; temperature, 25 °C; 80 mg of the sample was dissolved in 20 mL of the lower phase with four continuous sample injections in a single run. Fractions’ HPLC analysis results indicated that the three alkaloids (4.5 mg (yield 0.045%) 7-*epi*-javaniside, 2.9 mg (yield 0.03%) cadambine, and 2.5 mg (yield 0.025%) 3α-dihydrocadambine) were obtained in one step separation within 3 h (Figure 3). Compounds **2** and **3** were purified by prep-HPLC to obtain 3.2 mg (yield 0.032%) vincosamide and 3.4 mg (yield 0.034%) strictosamide. The HPLC analysis of each HSCCC fraction revealed that the purity values of compounds **1**, **4**, and **5** were 98.8%, 95.7%, and 98.5%, respectively. Compound **2** and compound **3** were separated into prep-HPLC to obtain purity of 99.2% and 95.7%. The HPLC chromatograms of the crude sample and all the target compounds were shown in Figure 4. Compounds **1**–**5** purity was recorded as 98.8%, 98.7%, 99.2%, 95.7%, and 98.5%, respectively, according to the HPLC peak-area percentages. This result showed that HSCCC can successfully and effectively separate compounds **1**–**5** from UR.

### 2.3. Structure Identification

The structure of the compound was determined by HR-ESI-MS and NMR. The MS and NMR data of compounds agree with published data.

**Compound 1** (Peak I in Figure 3, Figures S1–S3): Yellow powder, HR-ESI-MS *m*/*z* 515.2005 (M + H)^+^. ^1^H-NMR (600 MHz, CD_3_OD) *δ*: 4.08 (1H, dd, *J* = 11.4, 3.0 Hz, H-3), 3.96 (1H, td, *J* = 11.4, 7.3 Hz, H-5a), 3.84 (1H, brd, *J* = 12.4 Hz, H-5b), 2.48 (1H, dd, *J* = 12.8, 10.4 Hz, H-6a), 2.03 (1H, dd, *J* = 12.8, 7.3 Hz, H-6b), 6.90 (1H, d, *J* = 7.8 Hz, H-9), 7.03 (1H, td, *J* = 7.8, 1.2 Hz, H-10), 7.26 (1H, td, *J* = 7.8, 1.2 Hz, H-11), 6.97 (1H, d, *J* = 7.8 Hz, H-12), 1.40 (1H, dt, *J* = 12.6, 3.0 Hz, H-14a), 0.88 (1H, m, H-14b), 3.07 (1H, m, H-15), 2.54 (1H, ddd, *J* = 9.6, 6.8, 1.4 Hz, H-16), 5.42 (1H, d, *J* = 1.8 Hz, H-17), 7.41 (1H, d, *J* = 2.4 Hz, H-19), 5.30 (1H, dt, *J* = 16.8, 10.2 Hz, H-22), 5.07 (1H, dd, *J* = 17.1, 1.8 Hz, H-23a), 4.98 (1H, dd, *J* = 10.2, 1.8 Hz, H-23b), 4.64 (1H, d, *J* = 7.8 Hz, H-1′), 3.17 (1H, dd, *J* = 9.0, 7.8 Hz, H-2′), 3.35 (1H, m, *J* = 2.4 Hz, H-3′), 3.25 (1H, d, *J* = 2.4 Hz, H-4′), 3.27 (1H, d, *J* = 2.4 Hz, H-5′), 3.64 (1H, d, *J* = 12.0, 6.0 Hz, H-6′), ^13^C NMR (150 MHz, CD_3_OD) *δ*: 179.28 (C-2), 64.97 (C-3), 45.67 (C-5), 34.46 (C-6), 58.96 (C-7), 124.91 (C-9), 123.88 (C-10), 129.92 (C-11), 111.70 (C-12), 142.64 (C-13), 27.34 (C-14), 28.38 (C-15), 44.68 (C-16), 97.46 (C-17), 148.68 (C-19), 108.86 (C-20), 166.23 (C-21), 133.38 (C-22), 120.57 (C-23), 99.66 (C-1′), 74.84 (C-2′), 78.00 (C-3′), 71.69 (C-4′), 78.45 (C-5′), 62.80 (C-6′). Thus, the structure of **1** was defined as 7-*epi*-javaniside by comparison of its MS, ^1^H- and ^13^C-NMR data with literature data [17].

**Coumpound 2** (Peak II in Figure 3, Figures S4–S6): Yellow powder, HR-ESI-MS *m*/*z* 499.2058 (M + H)^+^. ^1^H-NMR (600 MHz, DMSO-*d*_6_) *δ*: 10.96 (1H, s, -NH), 4.91 (1H, m, H-3), 5.00 (1H, dd, *J* = 12.6, 5.4 Hz, H-5a), 2.89 (1H, dd, *J* = 12.6, 5.4 Hz, H-5b), 3.00 (1H, m, H-6a), 2.61 (1H, m, H-6b), 7.43 (1H, d, *J* = 8.4 Hz, H-9), 6.97 (1H, td, *J* = 8.4, 1.2 Hz, H-10), 7.07 (1H, td, *J* = 8.4, 1.2 Hz, H-11), 7.33 (1H, d, *J* = 8.4 Hz, H-12), 2.51 (1H, m, H-14a), 1.30 (1H, dd, *J* = 13.2, 11.4 Hz, H-14b), 2.73 (1H, m, H-15), 7.32 (1H, s, H-17), 5.34 (1H, dd, *J* = 17.2, 2.4 Hz, H-18a), 5.17 (1H, dd, *J* = 11.4, 2.4 Hz, H-18b), 5.48 (1H, dt, *J* = 17.4, 11.2 Hz, H-19), 2.68 (1H, m, H-20), 5.41 (1H, d, *J* = 2.4 Hz, H-21), 4.52 (1H, d, *J* = 7.8 Hz, H-1′), 3.04 (1H, m, H-2′), 3.18 (1H, m, H-3′), 3.12 (1H, m, H-4′), 3.21 (1H, m, H-5′), 3.67 (1H, m, H-6′a), 3.44 (1H, m, H-6′b); ^13^C-NMR (150 MHz, DMSO-*d*_6_) *δ*: 136.2 (C-2), 52.4 (C-3), 42.4 (C-5), 20.6 (C-6), 108.5 (C-7), 126.2 (C-8), 117.9 (C-9), 118.5 (C-10), 121.2 (C-11), 111.1 (C-12), 136.2 (C-13), 31.0 (C-14), 25.8 (C-15), 107.2 (C-16), 146.5 (C-17), 119.9 (C-18), 133.9 (C-19), 42.4 (C-20), 94.9 (C-21), 162.5 (C-22), 97.9(C-1′), 73.2 (C-2′), 77.2 (C-3′), 69.9 (C-4′), 76.5 (C-5′), 61.1 (C-6′). Thus, the structure of **2** was defined as vincosamide by comparison of its MS, ^1^H- and ^13^C-NMR data with literature data [18].

**Compound 3** (Peak III in Figure 3, Figures S7–S9): Yellow powder, HR-ESI-MS *m*/*z* 499.2063 (M + H)^+^. ^1^H-NMR (600 MHz, DMSO-*d*_6_) *δ*: 11.06 (1H, s, -NH), 5.01 (1H, brd, *J* = 4.2 Hz, H-3), 4.79 (1H, dd, *J* = 12.6, 5.4 Hz, H-5a), 2.99 (1H, m, H-5b), 2.80 (1H, m, H-6a), 2.61 (1H, m, H-6b), 7.35 (1H, d, *J* = 8.4 Hz, H-9), 6.97 (1H, td, *J* = 7.2, 0.6 Hz, H-10), 7.07 (1H, td, *J* = 7.2, 1.2 Hz, H-11), 7.33 (1H, d, *J* = 8.4 Hz, H-12), 2.51 (1H, m, H-14a), 1.89 (1H, td, *J* = 13.2, 5.4 Hz, H-14b), 2.60 (1H, m, H-15), 7.22 (1H, d, *J* = 1.8 Hz, H-17), 5.34 (1H, dd, *J* = 20.4, 1.8 Hz, H-18a), 5.32 (1H, m, H-18b), 5.58 (1H, dt, *J* = 17.4, 9.6 Hz, H-19), 2.58 (1H, m, H-20), 5.31 (1H, m, H-21), 4.42 (1H, d, *J* = 7.8 Hz, H-1′), 2.81 (1H, m, H-2′), 3.11 (1H, m, H-3′), 2.79 (1H, m, H-4′), 3.05 (1H, m, H-5′), 3.67 (1H, m, H-6′a), 3.42 (1H, m, H-6′b); ^13^C-NMR (150 MHz, DMSO-*d*_6_) *δ*: 134.8 (C-2), 52.7 (C-3),42.2 (C-5), 20.6 (C-6), 108.8 (C-7), 127.2 (C-8), 117.9 (C-9), 118.5 (C-10), 121.3 (C-11), 113.3 (C-12), 135.5 (C-13), 25.7 (C-14), 23.4 (C-15), 107.8 (C-16), 146.8 (C-17), 119.9 (C-18), 133.2 (C-19), 42.8 (C-20), 96.2 (C-21), 162.8 (C-22), 98.9 (C-1′), 72.6 (C-2′), 77.2 (C-3′), 69.8 (C-4′), 76.7 (C-5′), 61.2 (C-6′). Thus, the structure of **3** was defined as strictosamide by comparison of its MS, ^1^H- and ^13^C-NMR data with literature data [18].

**Compound 4** (Peak IV in Figure 3, Figures S10–S12): Yellow powder, HR-ESI-MS *m*/*z* 545.2113 (M + H)^+^. ^1^H-NMR (600 MHz, CD_3_OD) *δ*: 2.82 (1H, m, H-5α), 3.14 (1H, m, H-5β), 2.80 (2H, m, H-6), 7.47 (1H, d, *J* = 8.4 Hz, H-9),7.01 (1H, t, *J* = 7.1 Hz, H-10), 7.11 (1H, t, *J* = 7.5 Hz, H-11), 7.33 (1H, d, *J* = 7.8 Hz, H-12), 2.05 (1H, m, H-14α), 2.08 (1H, m, H-14β), 2.80 (1H, m, H-15), 7.57 (1H, s, H-17), 3.03 (1H, dd, *J* = 10.8, 7.8 Hz, H-18α), 3.49 (1H, brd, *J* = 10.2 Hz, H-18β), 4.92 (1H, m, H-19), 1.73 (1H, m, H-20), 5.84 (1H, d, *J* = 9.0 Hz, H-21), 3.65 (3H, s, -OCH_3_), 4.80 (1H, d, *J* = 7.8 Hz, H-1′), 3.31~3.40 (4H, m, H-2′~5′), 3.61 (1H, dd, *J* = 12.0, 6.6 Hz, H-6′α), 3.87 (1H, dd, *J* = 12.0, 2.4 Hz, H-6′β); ^13^C-NMR (150 MHz, CD_3_OD) *δ*: 133.2 (C-2), 93.0 (C-3), 53.8 (C-5), 22.8 (C-6), 111.6 (C-7), 126.9 (C-8), 120.2 (C-9), 119.8 (C-10), 123.4 (C-11), 112.5 (C-12), 138.60 (C-13), 43.1 (C-14), 26.9 (C-15), 111.3 (C-16), 154.4 (C-17), 59.5 (C-18), 74.5 (C-19), 41.1 (C-20), 97.7 (C-21), 168.9 (C-22), 51.8 (-OCH_3_), 101.7 (C-1′), 74.9 (C-2′), 78.5 (C-3′), 71.7 (C-4′), 78.1 (C-5′), 62.9 (C-6′). Thus, the structure of **4** was defined as cadambine by comparison of its MS, ^1^H- and ^13^C-NMR data with literature data [19].

**Compound 5** (Peak V in Figure 3, Figures S13–S15): Yellow powder, HR-ESI-MS *m*/*z* 547.2261 (M + H)^+^. ^1^H-NMR (600 MHz, CD_3_OD) *δ*: 3.84 (1H, m, H-3), 2.80 (2H, m, H-6), 7.39 (1H, d, *J* = 7.8 Hz, H-9), 6.98 (1H, t, *J* = 7.4 Hz, H-10), 7.05 (1H, t, *J* = 7.6 Hz, H-11), 7.30 (1H, d, *J* = 8.0 Hz, H-12), 2.38 (1H, d, *J* = 14.6 Hz, H-14a), 1.79 (1H, dt, *J* = 14.6 Hz, 10.4Hz, H-14b), 3.08 (1H, m, H-15), 7.57 (1H, s, H-17), 3.29 (1H, m, H-18a), 2.93 (1H, m, H-18b), 4.35 (1H, dd, *J* = 7.5Hz, 5.3Hz, H-19), 2.09 (1H, dt, *J* = 9.5 Hz, 5.9Hz, H-20), 5.61 (1H, d, *J* = 9.1 Hz, H-21), 3.82 (3H, s, -OCH_3_), 4.82 (1H, d, *J* = 7.9 Hz, H-1′), 3.31~3.42 (4H, m, H-2′-5′), 3.86(1H, m, H-6′a), 3.68(1H, m, H-6′b); ^13^C-NMR (150 MHz, CD_3_OD) *δ*: 136.1 (C-2), 64.1 (C-3), 56.1 (C-5), 23.6 (C-6), 108.8 (C-7), 128.3 (C-8), 118.7 (C-9), 119.9 (C-10), 122.2 (C-11), 112.2 (C-12), 138.4 (C-13), 37.6 (C-14), 34.2 (C-15), 111.4 (C-16), 153.7 (C-17), 59.6 (C-18), 66.8 (C-19), 44.8 (C-20), 97.7 (C-21), 169.6 (C-22), 52.2 (-OCH3), 100.9 (C-1′), 74.7 (C-2′), 78.5(C-3′), 71.2 (C-4′), 78.1 (C-5′), 62.5 (C-6′), Thus, the structure of **5** was defined as 3α-dihydrocadambine by comparison of its MS, 1H- and 13C-NMR data with literature data [20].

### 2.4. Cholinesterase Inhibitory Activity

Activity test results showed that the crude extract had strong cholinesterase inhibitory activity and obtained five alkaloids with cholinesterase inhibitory activity through further separation, which confirmed the feasibility of the experimental research method. Compounds **1**–**5** showed strong acetyl-cholinesterase and butyryl-cholinesterase inhibitory activities. Among them, compound **1** exhibited an inhibitory effect close to that of the positive drug, suggesting that this compound can be used as a lead compound for cholinesterase inhibitors (Table 2).

### 2.5. Molecular Docking Result

**Figure 5:**
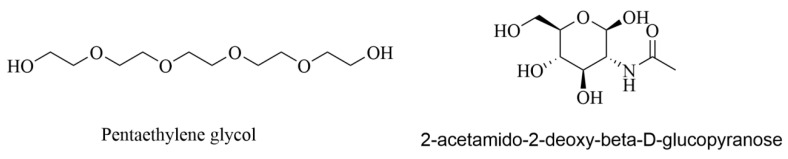
Chemical structure of AchE and BuchE native ligands.

Mostly, molecular docking studies are applied to drug development, including the discovery of novel AchE/BuchE inhibitors. Furthermore, molecular docking also predicts the binding orientation of the ligands to the active sites of AchE/BuchE frequently. In this process, AutoDock Vina has proven to be a powerful tool for evaluating the binding efficiency between ligands and protein targets [21]. Therefore, in the present study, AutoDock Vina v1.1.3 was applied to simulate the molecular recognition process between AchE/BuchE and the compounds separated above from UR, and the binding energies were calculated simultaneously (Table 3). The native ligands (Figure 5) of AchE and BuchE are pentaethylene glycol (C_10_H_22_O_6_) and 2-acetamido-2-deoxy-β-D-glucopyranose (C_8_H_15_NO_6_).

**Figure 6:**
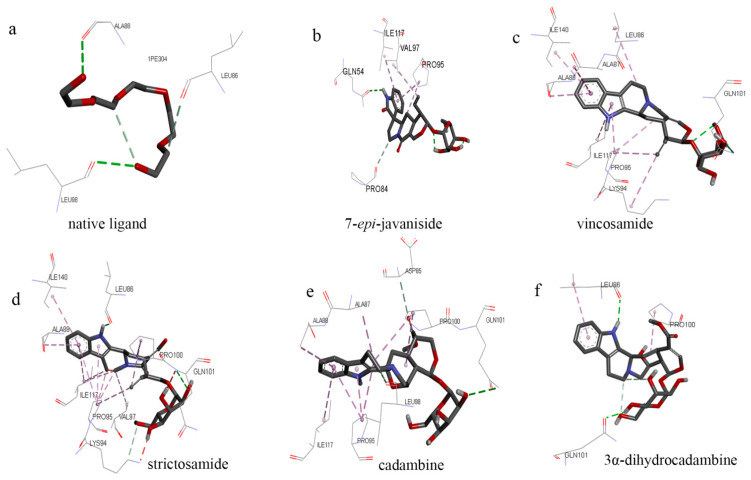
Three-dimensional (3D) figures of molecular docking between compounds **1**–**5** and AchE ((**a**), native ligand; (**b**–**f**), compounds **1**–**5**).

The affinity energy of the identified compounds ranges from −6.0 to −7.6 kcal/mol, a more desirable result than organic ligand (−3.9 kcal/mol), indicating that those five compounds may have the potential to be AchE inhibitors. As shown in Figure 6, the organic ligand interacts with Ala88, Leu 98, and Leu86 residues, which also indicates that these residues may be in the main AchE active sites. In this result, compared with other compounds, compounds **2** (−6.3 kcal/mol) and **4** (−6.0 kcal/mol) show higher affinity energy. Based on the phenomenon, one of the reasons may be that they form interaction with AchE through Ala87, Ala88, Ile117, and Gln101, respectively. Compounds **1** (−7.6 kcal/mol) and **3** (−7.5 kcal/mol) show more stable binding, which may be related to the Val97, Pro95, and Ile117 residue sites. These residues may be potential sites for inhibiting AchE activity. In the future, mutation experiments can be carried out on their amino acid sites to verify whether they are active sites. Compared with compound **4**, compound **5** has a better binding ability, suggesting that it has a better AchE inhibition ability. In terms of binding sites, although compound **5** has fewer binding sites, both have the same binding sites Pro100 and Gln101 compared with compound **4**. Therefore, from the structural point of view, the dihydrogenation of compound 5 makes its ability to bind sites more efficient. In addition, by comparing the interaction residue sites of all compounds with AchE, it was found that Ala88, Leu86, Pro95, Pro100, Val97, and Ile117 can be used as the main targets for the follow-up study of AchE inhibitors.

**Figure 7:**
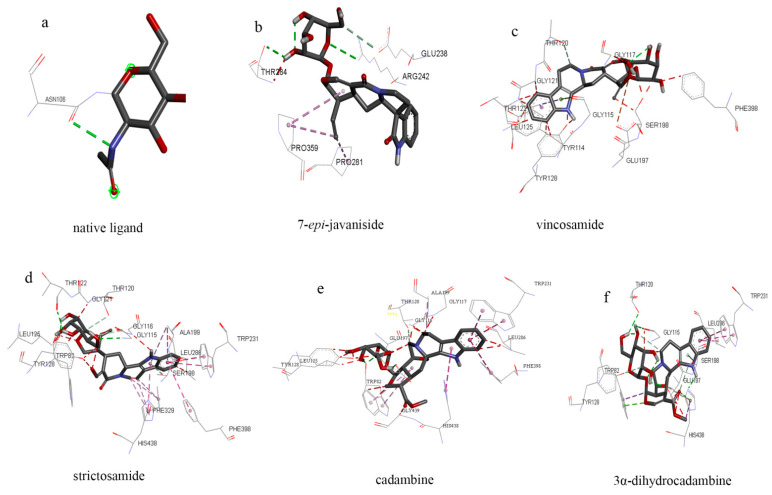
Three-dimensional (3D) figures of molecular docking between compounds **1**–**5** and BuchE ((**a**), native ligand; (**b**–**f**), compounds **1**–**5**).

The intermolecular interaction between compounds **1**–**5** and BuchE is shown in Figure 7. Compounds **2** (−8.4 kcal/mol) and **4** (−8.7 kcal/mol) show more stable binding ability, which may be related to amino acid residues, Trp32, Thr120, Gly117, Leu125, and Phe398, which were involved in the interaction. However, compound **1** also showed good binding ability (−8.1 kcal/mol), and the binding sites (Glu238, Arg242, Thr284, Pro359, and Pro281) were different from compounds **2** and **4**. This suggests that compound **1** may have a specific mechanism of action, and the emergence of Arg242 means that this specific mechanism may be related to this amino acid. Although compounds **3** (−7.6 kcal/mol) and **5** (−7.5 kcal/mol) show poor BuchE binding ability, there were more hydrogen bonds in the form of interaction. These hydrogen bonds overtly strengthened the interaction and served as anchors for binding the inhibitor in the active site. This can be speculated as it may be affected by the intermolecular force or hydrophobic interactions, which make the binding energy unstable. In the in vitro experimental verification results, compound **1** showed better inhibition of AchE and BuchE, which were further supported by the molecular docking result. However, the simulation results of compounds **2** and **4** combined with BuchE were inconsistent with those in vitro. This is not surprising, because the results of molecular docking are not always consistent with experiments in vitro. In the process of molecular docking, all possible spatial structures of molecules will be simulated, but not all possible spatial structures can exist stably, which may be the main reason for the different results between activity experiments and molecular docking. It is speculated that it may have some spatial structures with good AchE and BuchE inhibitory activity, but these structures can’t exist stably. The molecular docking results and in vitro experiments of compound **1** may have a spiro ring structure, which makes its spatial structure diverse and has good activity. The above experimental results show that molecular docking can be used as an efficient screening tool for AchE and BuchE inhibitors.

## 3. Experimental

### 3.1. Chemicals and Reagents

For the preparation of crude extract and counter-current chromatography (CCC) separation of ethanol, ethyl acetate, methanol, methyl tert-butyl ether, and *n*-butanol were analytical grade (Sinopharm Chemical Reagent Co., Ltd., Shanghai, China). HPLC-grade methanol and acetonitrile were purchased from Shanghai, China (Sinopharm Chemical Reagent Co., Ltd., Shanghai, China). Acetyl-cholinesterase was from electric eel, butyryl-cholinesterase was from equine serum, and acetylthiocholine iodide was purchased from Sigma-Aldrich (Sigma-Aldrich company, St. Louis, MO, USA). Tacrine and 5, 5′-dithio-bis-(2-nitrobenzoic acid) were provided by Macklin (Macklin Biochemical Technology Co., Ltd., Shanghai, China). AB-8 macroporous resin was purchased from Yuanye (Yuanye Biochemical Technology Co., Ltd., Shanghai, China).

UR (1.0 kg) was collected from Zhuzhou, Hunan Province, China in March 2018, and identified by Professor Li Feng of Shandong University of Traditional Chinese Medicine. A voucher specimen (180301) had been deposited in the herbarium of Shandong University of Traditional Chinese Medicine.

### 3.2. Apparatus

The HSCCC equipment was TBE-300C (Shanghai, Tauto Biotech, China) with three multilayer coil separation columns of 300 mL (diameter of the PTFE tube as 2.6 mm) as well as a 20 mL manual sample loop. The HSCCC apparatus was equipped with three other instrument modules, including a TBP-5002s constant-flow pump, a TBD-2000 UV detector (Tauto Biotechnique, Shanghai, China), and a DC-0506 low constant temperature bath (Tauto Biotechnique, Shanghai, China) to maintain the temperature at 25 °C. HPLC separation was performed on a 1260 LC system (Agilent Technologies, Santa Clara, CA, USA) consisting of a quaternary pump, an online degasser, a diode array detector, an auto plate sampler, and a thermostatically controlled column compartment. Prep-HPLC FL-H050G (Agela Technologies, Tianjin, China). An AMR-100 automatic enzyme label analyzer (Allsheng Instruments Co., Ltd., Hangzhou, China) was used to determine the absorbance. T5-100C A gas bath constant temperature oscillator (Kuang Bei Industrial Co., Ltd., Shanghai, China) provided constant temperature incubation conditions. NMR spectra were obtained from a Bruker Avance III 600 MHz spectrometer (Bruker corporation, Saarbrucken, Germany). The chemical shift values are reported as *δ* in ppm relative to tetramethylsilane (TMS) and the coupling constants (*J*) are in hertz (Hz). HR-ESI-MS spectra were recorded on Thermo Scientific Q Exactive UHMR-Orbitrap (Thermo Fisher Scientific company, Waltham, MA, USA).

### 3.3. Preparation of Crude Extract

The dried stems of UR (1.0 kg) were crushed and extracted three times using 5 L of C_2_H_5_OH:water (7:3, *v*/*v*) at 25 °C for 15 days, and the extraction was repeated three times; then dried under reduced pressure to obtain ethanol extract (100 g) and suspended in water and extracted with *n*-butanol (1.0 L).

The *n*-butanol soluble fraction (22.3 g) was first separated over a macroporous adsorbent resin column (AB-8). The column was eluted with water:ethanol (90:10, 70:30, 50:50, 30:70, 10:90, *v*/*v*), yielding five fractions. Then, the 30% ethanol eluate (0.8 g) was ready to be further separated by HSCCC.

### 3.4. Selection of Solvent System

The selection of the solvent system is based on the partition coefficients (*K*-values). The *K*-values of the target compounds in the crude alkaloid extract from the UR were determined by HPLC. Five milliliters of each phase of the equilibrated two-phase solvent system was added to approximately 10 mg of the crude alkaloid extract and were shaken vigorously for 1 min. After the phases had fully separated, 1 mL of each layer was removed and dried in a stream of nitrogen. The residues were dissolved in 1 mL of methanol and analyzed by HPLC. The *K*-values of the target compounds were calculated according to Equation (1), where A_U_ and A_L_ were the peak areas of the target compound in the upper and lower phases, respectively.

*K* = A_U_/A_L_ (1)

### 3.5. Preparation of the Solvent System and Sample Solutions

For the HSCCC solvent system, a two-phase system, consisting of ethyl acetate:*n*-butanol:water (1:4:5, *v*/*v*/*v*), was placed into a separating funnel. After shaking vigorously, the solution was allowed to stand for several minutes and was separated into two phases for the experiment. The upper phase was the stationary phase, while the lower phase was the mobile phase. For this HSCCC sample, 80 mg of crude extract alkaloid was dissolved in 20 mL isometric upper and lower phase.

### 3.6. HSCCC Separation Procedure

In the separation procedure, the multilayer coiled column was entirely filled with the upper phase as the stationary phase at first. Then, the lower phase as mobile phase was pumped into the head end of the column at a flow rate of 2.0 mL/min, while the apparatus was rotated at 800 rpm. The temperature of the low-temperature constant temperature bath was set to 25 °C. After a clear mobile phase eluted at the tail outlet, which means hydrodynamic equilibrium was established, the sample solution was injected through the sample port. A UV detector was used to monitor the effluent from the tail end of the column continuously at 220 nm. Each peak fraction was collected according to the chromatogram. The retention of the stationary phase was calculated from the volume of the stationary phase collected from the column before the sample was injected.

### 3.7. Further Separation by Prep-HPLC

In Figure 3, we can see that compounds **1**, **4**, and **5** reached the baseline separation state with neighboring compounds. In this case, the fractions can be directly taken out and tested for purity by HPLC and further identification. Compounds **2** and **3** did not reach the baseline separation, and the two peaks inevitably crossed, resulting in reduced compound purity. The mixture of compounds **2** and **3** from HSCCC was dried on a rotary evaporator at a temperature of 55 °C and dissolved with methanol and water (4:6, *v*/*v*). A ReproSil 100 C_18_ (250 mm × 10 mm, 5 µm) was used for the separation procedure. The mobile phase was composed of water (A) and methanol (B), and a 45% B isocratic elution in 40 min was used with a detection wavelength of 220 nm. The flow rate was 3.0 mL/min and the injection volume was 0.5 mL.

### 3.8. HPLC Analysis Peak

HPLC analysis of the crude sample and each peak fraction were performed as follows: a ZORBAX-SB-C_18_ column (5 µm, 150 × 4.6 mm) was used with gradient elution at a flow rate of 1.0 mL/min. The mobile phase was water (contained 0.2% ammonia, A) and acetonitrile (B) (0–35 min, 10–40% B). The work of structure identification was carried out by HR-ESI-MS and NMR.

### 3.9. Cholinesterase Inhibitory Activity

The Ellman [22] method was used to determine the inhibitory activity of acetyl-cholinesterase and butyryl-cholinesterase of each monomer compound. Briefly, 150 µL of PBS buffer solution (pH = 7.4), 10 µL of the tested compound solution, and 20 µL of acetyl-cholinesterase or butyryl-cholinesterase solution (concentration of 0.2 U/mL) were transferred to a 96-well plate and placed in a gas bath constant temperature oscillator at 37 °C for 15 min. After that, 10 µL of 5, 5′-dithio-bis-(2-nitrobenzoic acid) solution (DNTB, 2 mmol/L) and 10 µL of acetylthiocholine iodide solution were added in sequence (AICI, 10 mmol/L), mixed well, and after 30 min, the absorbance of each well was measured and recorded at a wavelength of 412 nm, repeated three times and the average was taken. Tacrine was used as a positive control and DMSO was used as a negative control. Then, 10 µL PBS was added to the blank group to exclude other irrelevant factors from affecting the experimental results and ensure the rationality of the experimental results. The inhibition rate at each concentration according to the following Equation (2) and the half inhibitory concentration (IC_50_ value) of AchE and BuchE were calculated.

Inhibition Rate = (A_negative control group_ − A_test group_/A_negative control group_ − A_blank group_) × 100% (2)

### 3.10. Molecular Docking

Molecular docking was chosen for understanding the mode of interactions between target compounds and AchE/BuchE. The docking program was performed by using Auto Dock Vina v1.1.3. Firstly, the structures of these compounds were saved as a docking ligand in PDB format, and the energy of ligand molecules was minimized by using Chem 3D 20.0. The X-ray crystal structures of AchE (PDB ID: 6U37) and BuChE (PDB ID: 6R6W) were download from the RSCB database (https://www.pdbus.org/, accessed on 21 August 2019). Secondly, hydrogen atoms were added to the protein structure to ensure the correct protonation states. Then, all ligands including the protein were converted to PDBQT format. Concurrently, the docking analysis was performed in a grid map of 18 × 20 × 20 with the spacing of 1 Å centered on the active site of AchE/BuchE. Finally, the docking results were visualized using PyMOL software (https://www.pymol.org/, accessed on 21 August 2019), and docking results were evaluated by the affinity value.

## 4. Conclusions

In our study, a new method, using a two-phase solvent system composed of ethyl acetate:*n*-butanol:water (1:4:5, *v*/*v*/*v*) combined with the further purification of the prep-HPLC, was successfully established to isolate five high polar alkaloids from the UR crude extract. After HPLC analysis, the purity of them was determined to be 98.8%, 98.7%, 99.2%, 95.7%, and 98.5%, respectively. Compounds **1**, **4**, and **5** were firstly isolated from the plant of UR. In addition, the enzyme activity experiment and molecular docking showed that the five alkaloids isolated have varying degrees of cholinesterase inhibitory activity, indicating the feasibility of this method for targeted preparation of isolated cholinesterase inhibitors. Compounds **2** and **3** are isomers of each other. HSCCC was successfully used to separate isomers in UR, which provided a theoretical reference for the subsequent separation of isomers. In short, the current study advances our understanding of the chemical composition and biological effects of UR. Furthermore, the above compounds, especially compound **1**, can be used for in-depth studies for their inhibition of AchE/BuchE and further accelerate the design of drugs for AD treatment.

## Figures and Tables

**Table 1:** The *K* values of target compounds in HSCCC separation with different solvent systems.

Slovent Systems	Ratio (*v*/*v*/*v*)	*K*				
		1	2	3	4	5
Methyl-tert-butyl Ether:acetonitrile:water	4:1:5	1.92	2.34	2.36	3.15	4.72
*n*-butanol:water	1:1	0.03	0.07	0.09	0.43	0.36
Ethyl acetate:*n*-butanol:water	4:1:5	0.07	0.35	0.39	0.74	1.53
Ethyl acetate:*n*-butanol:water	4:0.5:5	0.05	0.22	0.24	0.82	0.96
Ethyl acetate:*n*-butanol:water	3:2:5	0.11	0.71	0.82	0.95	1.11
Ethyl acetate:*n*-butanol:water	1:4:5	0.21	0.53	0.68	1.03	1.95
Ethyl acetate:*n*-butanol:water	1:5:5	0.69	1.16	1.18	1.74	2.14

**Table 2:** Cholinesterase activity of compound **1**–**5**.

Samples	IC_50_ Value (μmol/L)	
	Acetylcholinesterase	Butyrylcholinesterase
Crude extract	8.17 ± 0.17	22.53 ± 1.13
1	2.85 ± 0.50	2.13 ± 0.10
2	12.4 ± 0.86	23.18 ± 0.14
3	46.57 ± 0.58	6.47 ± 0.72
4	26.12 ± 2.12	30.69 ± 0.69
5	37.01 ± 1.57	33.34 ± 0.51
Tacrine	4.39 ± 0.80	3.25 ± 1.86

**Table 3:** Molecular docking of compounds **1**–**5** with AchE and BuchE.

Ligands	AchE (kcol/mol)	BuchE (kcol/mol)
Native ligand (AchE)	−3.9	---
Native ligand (BuchE)	---	−7.4
Compound 1	−7.6	−8.1
Compound 2	−6.3	−8.4
Compound 3	−7.5	−7.6
Compound 4	−6.0	−8.7
Compound 5	−6.9	−7.5

“---”: no binding.

## Data Availability

All spectral data about the article are available online.

## Supplementary Materials

The following supporting information can be downloaded online: https://www.mdpi.com/article/10.3390/molecules27062013/s1, Figures S1–S10: NMR spectra of compounds **1**–**5**; Figures S11–S15: HRMS spectra of compounds **1**–**5**.

## Sample Availability

Samples of extract crude are available from the authors.
