# Supplementary material for: Bioactivity-Guided Separation of Anti-Cholinesterase Alkaloids from Uncaria rhynchophlly (Miq.) Miq. Ex Havil Based on HSCCC Coupled with Molecular Docking
Source: Molecules. 2022 Mar 21;27(6):2013. doi: 10.3390/molecules27062013 (PMC8949104; doi:10.3390/molecules27062013)
Supplement: Supplementary file 1 [file molecules-27-02013-s001.zip › molecules-1631050-supplementary.pdf]

Supplementary Materials

Figure S1 The ^{1}H-NMR spectrum of compound **1**
Figure S2 The ^{13}C-NMR spectrum of compound **1**
Figure S3 The ^{1}H-NMR spectrum of compound **2**
Figure S4 The ^{13}C-NMR spectrum of compound **2**
Figure S5 The ^{1}H-NMR spectrum of compound **3**
Figure S6 The ^{13}C-NMR spectrum of compound **3**
Figure S7 The ^{1}H-NMR spectrum of compound **4**
Figure S8 The ^{13}C-NMR spectrum of compound **4**
Figure S9 The ^{1}H-NMR spectrum of compound **5**
Figure S10 The ^{13}C-NMR spectrum of compound **5**
Figure S11 The HR-ESI-MS spectrum of compound **1**
Figure S12 The HR-ESI-MS spectrum of compound **2**
Figure S13 The HR-ESI-MS spectrum of compound **3**
Figure S14 The HR-ESI-MS spectrum of compound **4**
Figure S15 The HR-ESI-MS spectrum of compound **5**

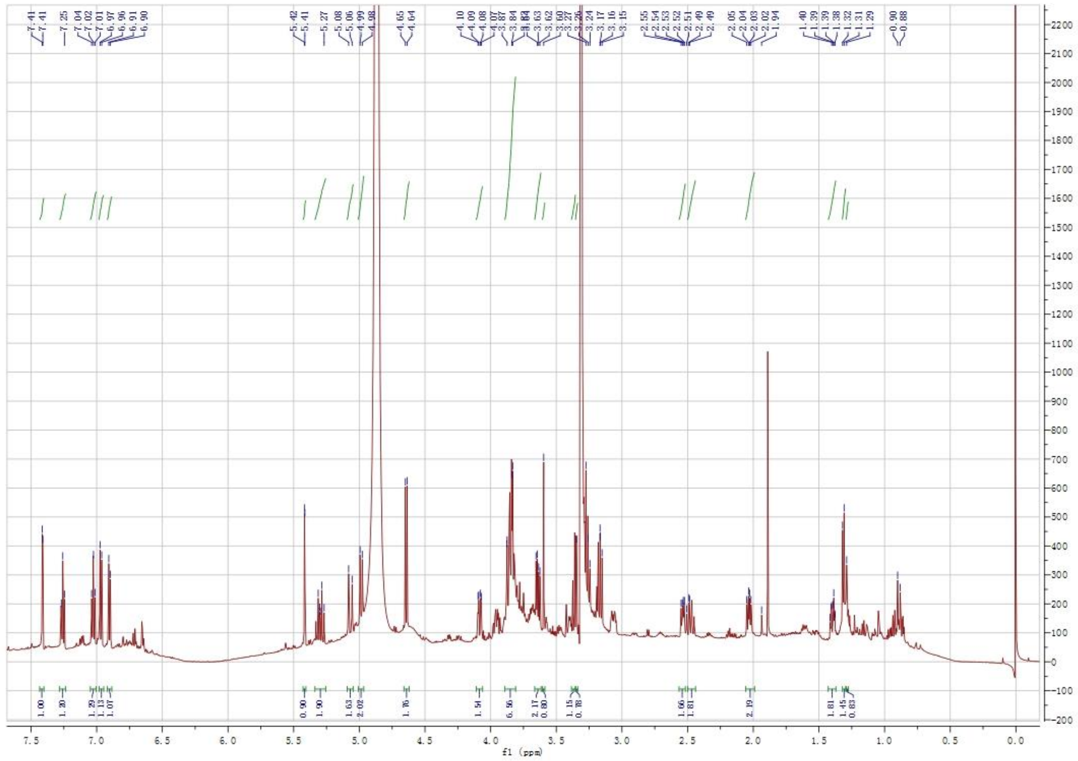

Figure S1 The ^{1}H-NMR spectrum of compound **1**

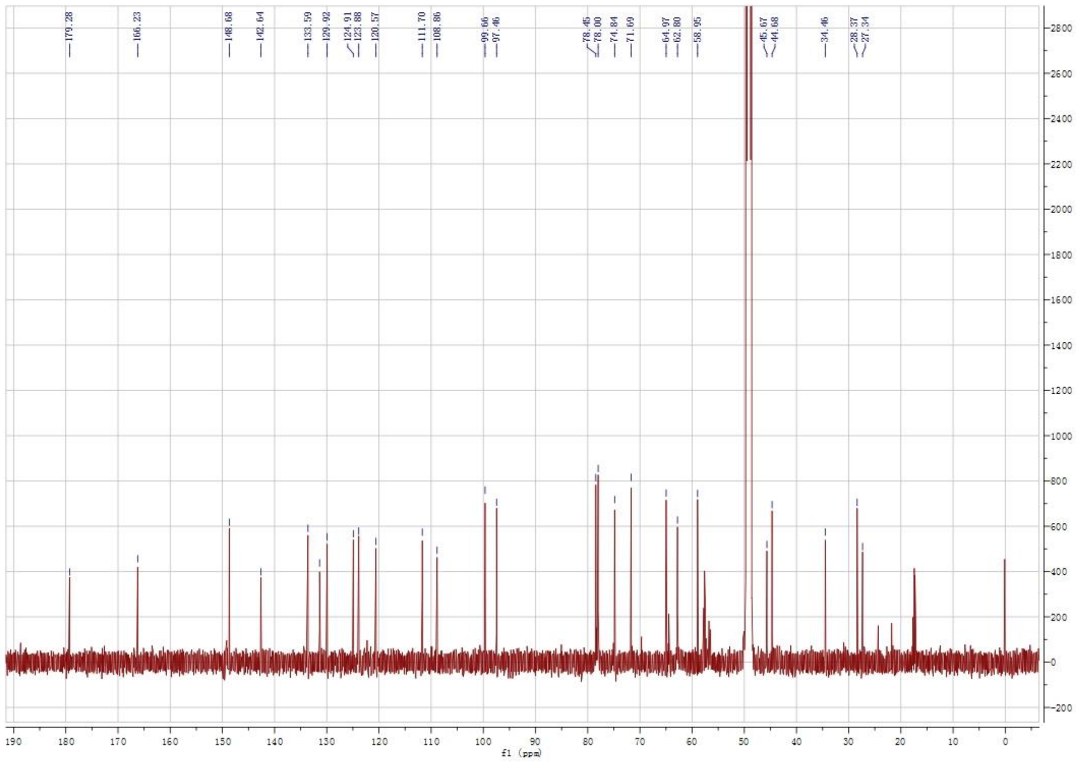

Figure S2 The ^{13}C NMR spectrum of compound **1**

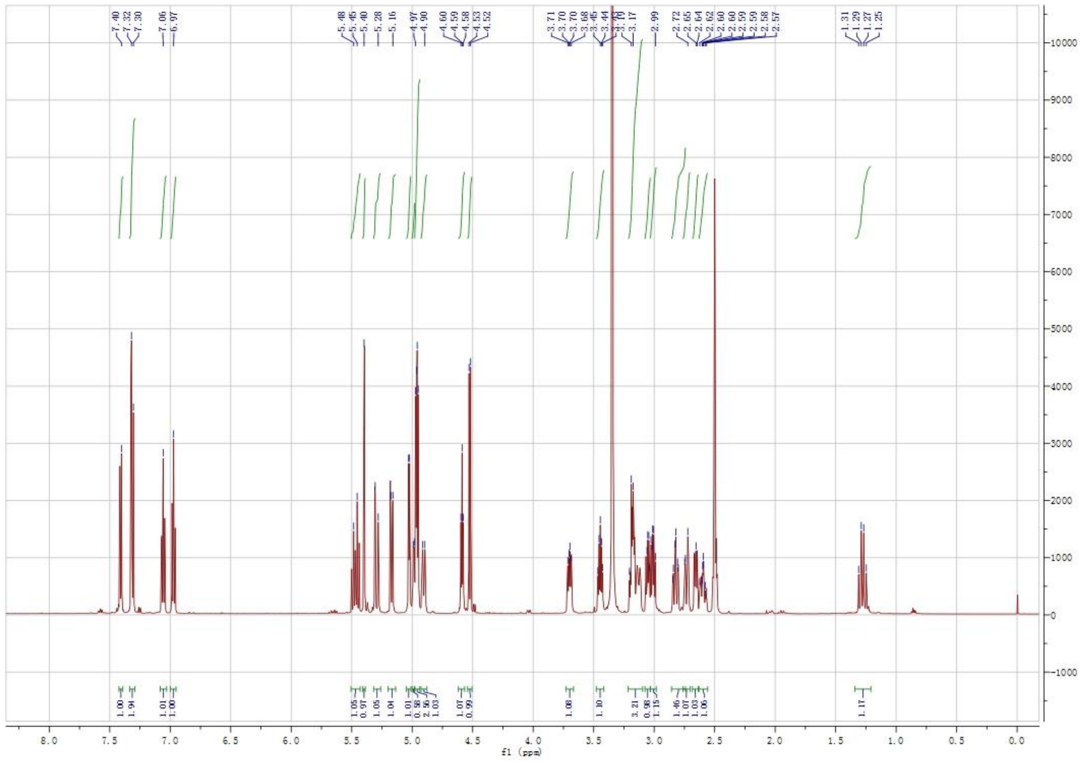

Figure S 3 The ^{1}H NMR spectrum of compound **2**

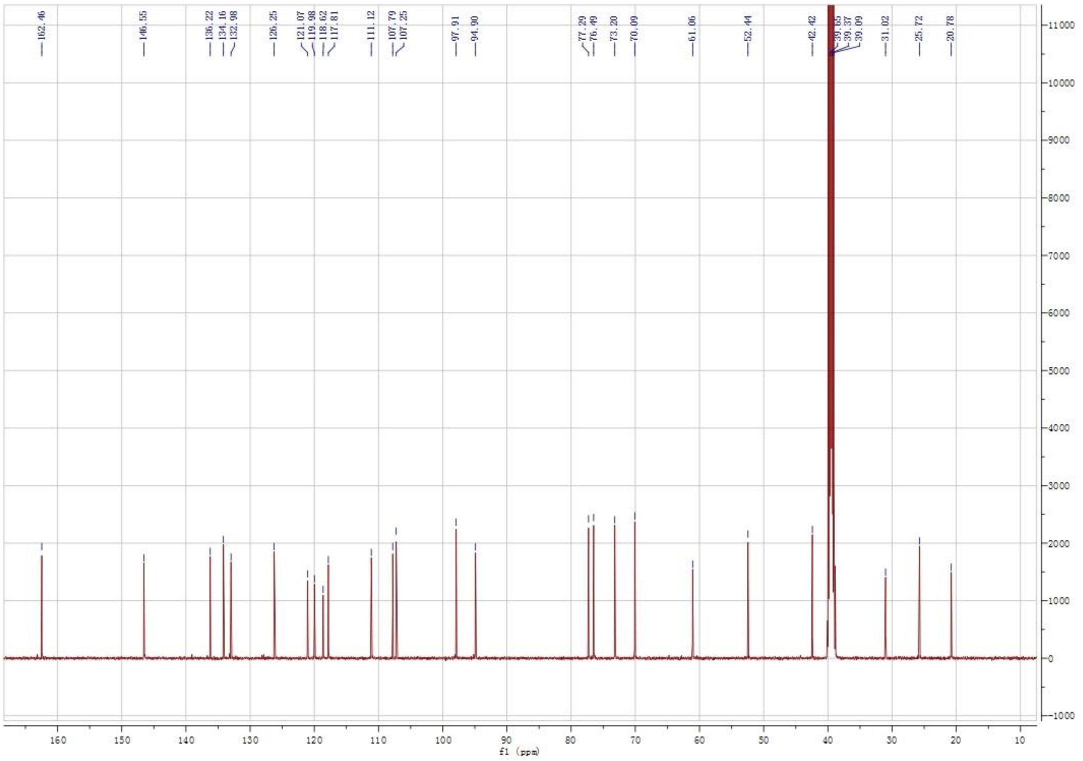

Figure S4 The ^{13}C NMR spectrum of compound **2**

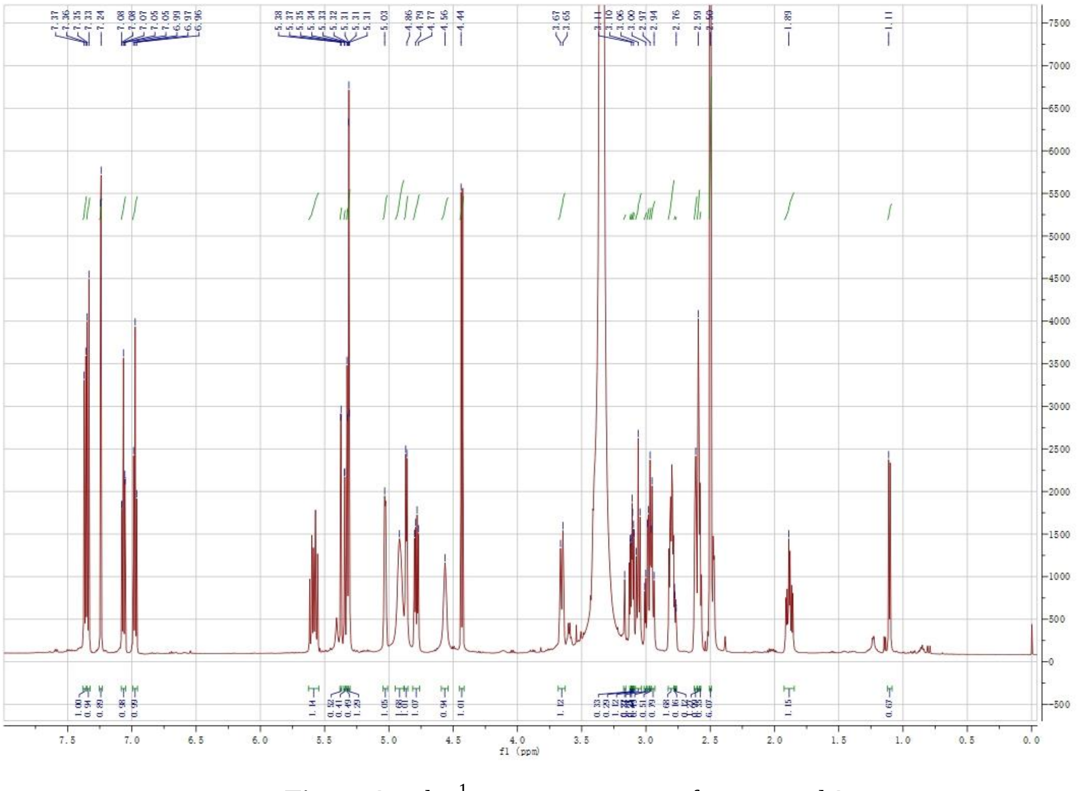

Figure S5 The ^{1}H NMR spectrum of compound **3**

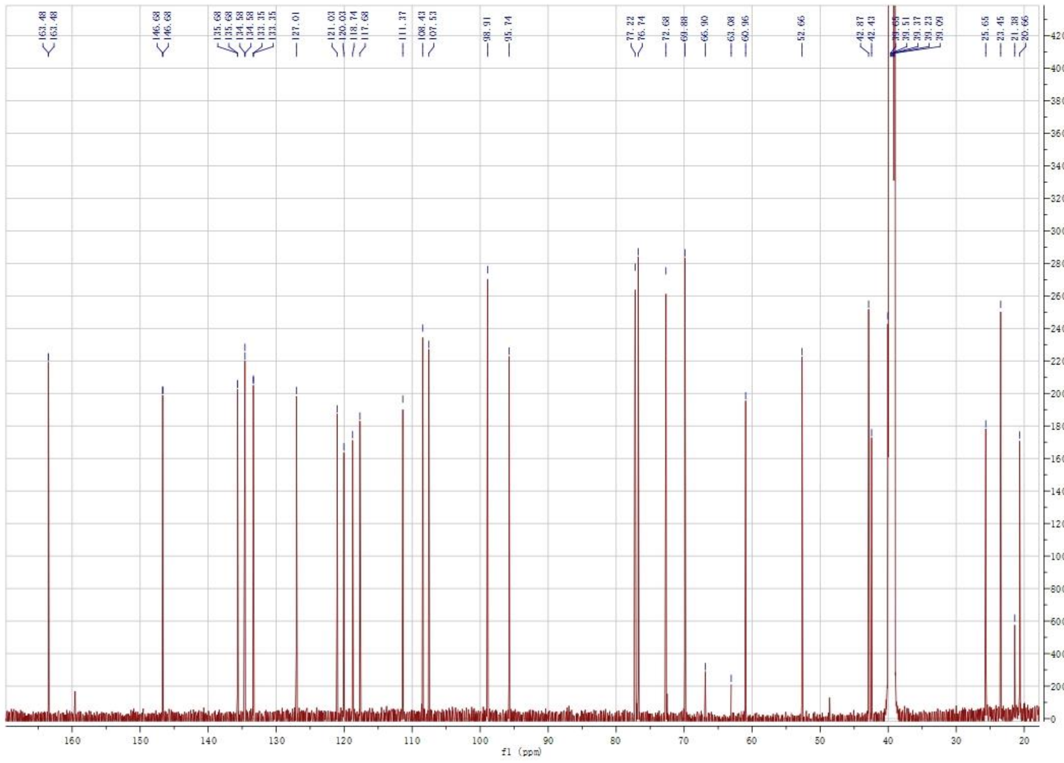

Figure S6 The ^{13}C NMR spectrum of compound **3**

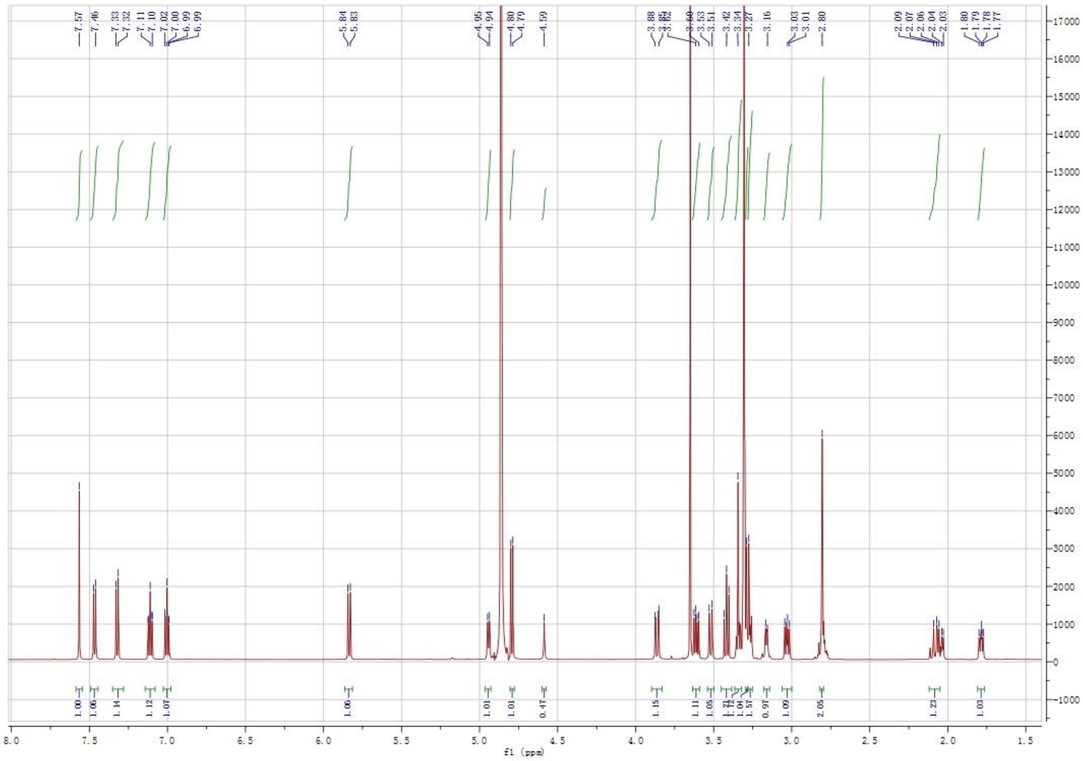

Figure S7 The ^{1}H NMR spectrum of compound **4**

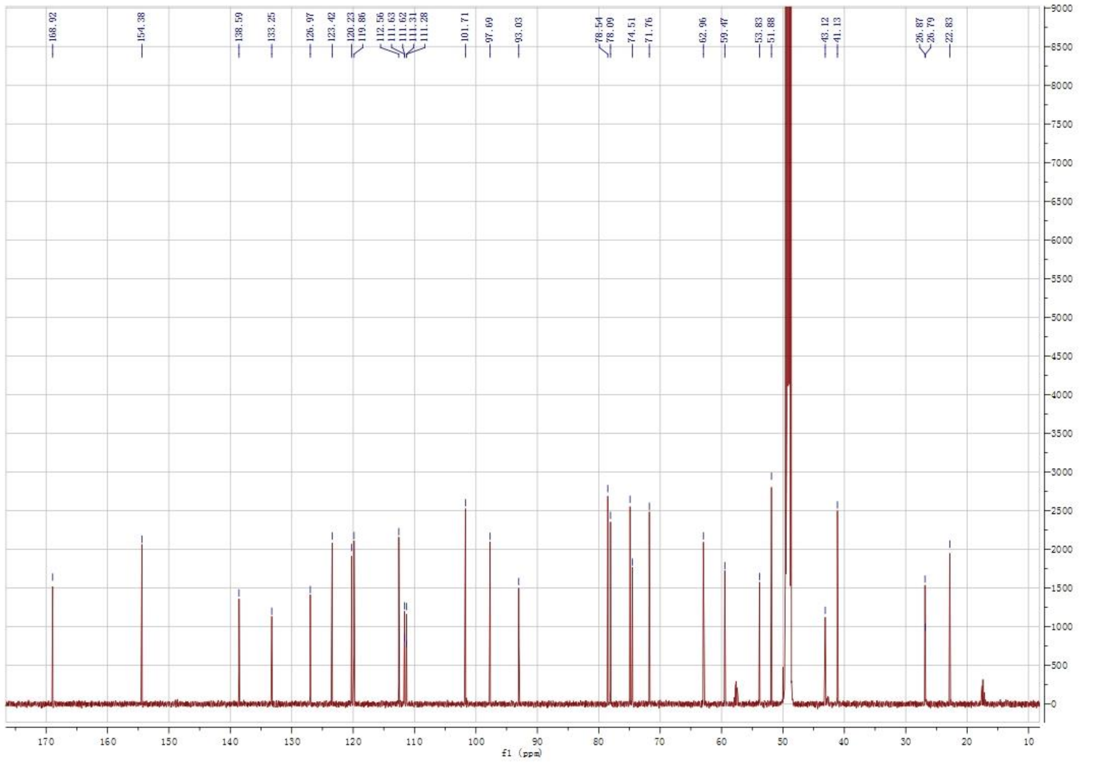

Figure S8 The ^{13}C NMR spectrum of compound **4**

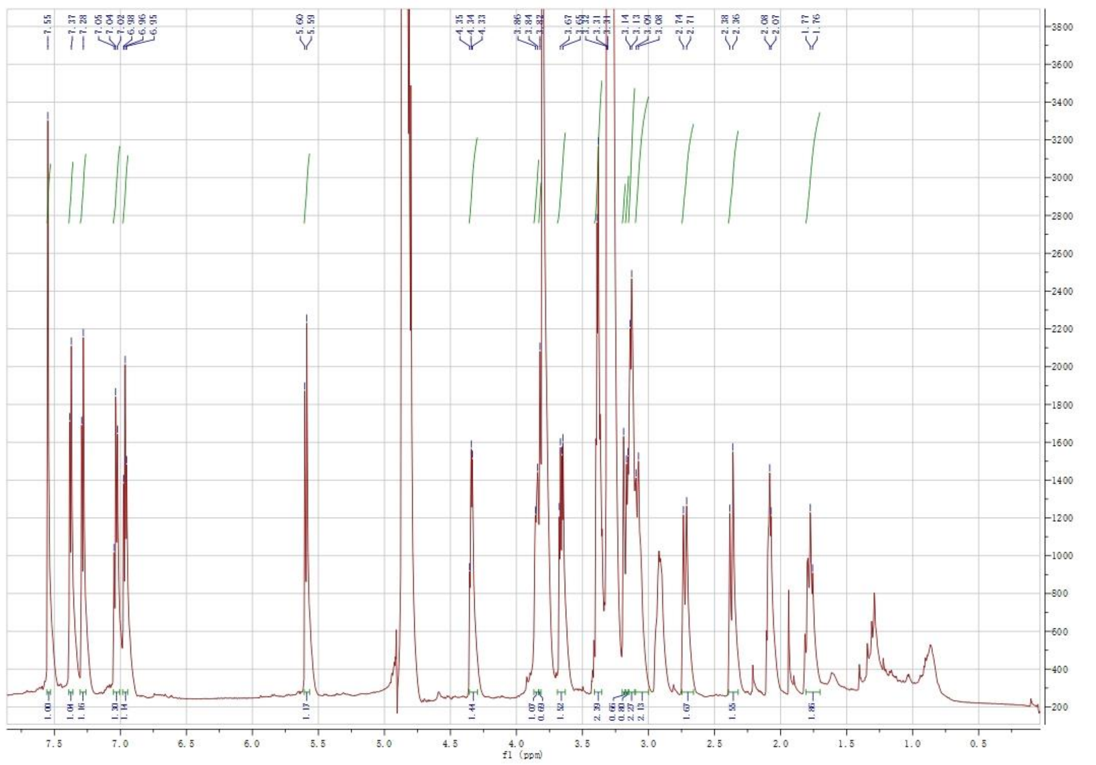

Figure S9 The ^{1}H NMR spectrum of compound **5**

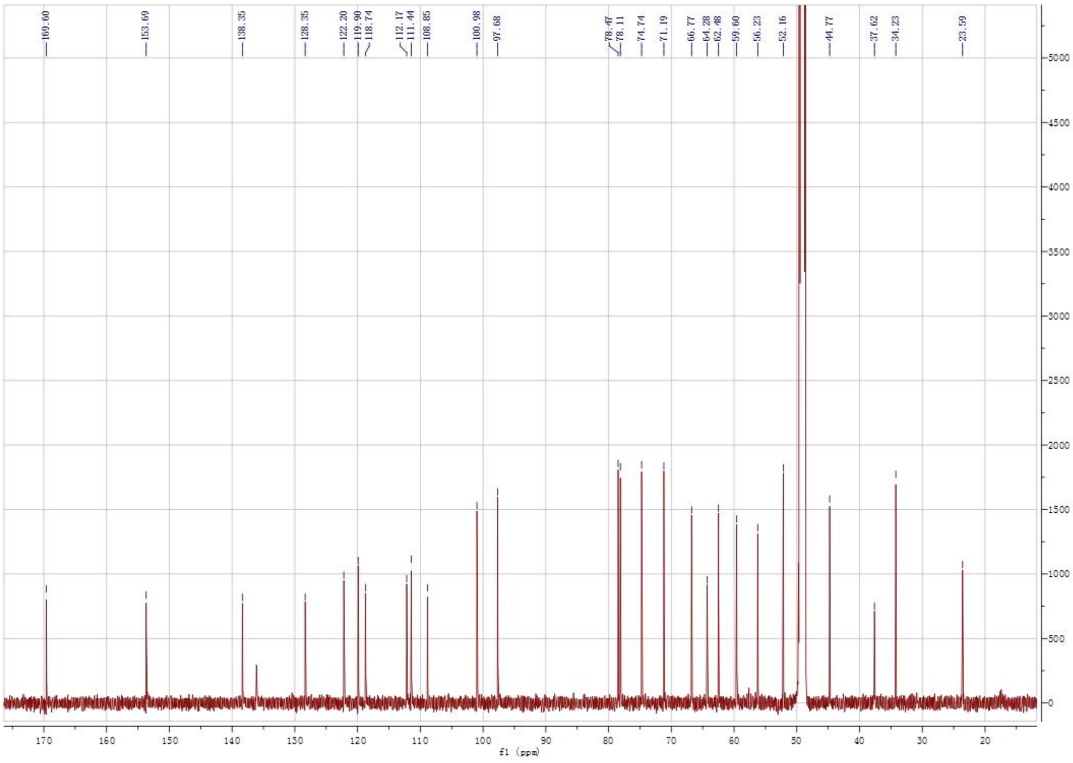

Figure S10 The ^{13}C NMR spectrum of compound **5**

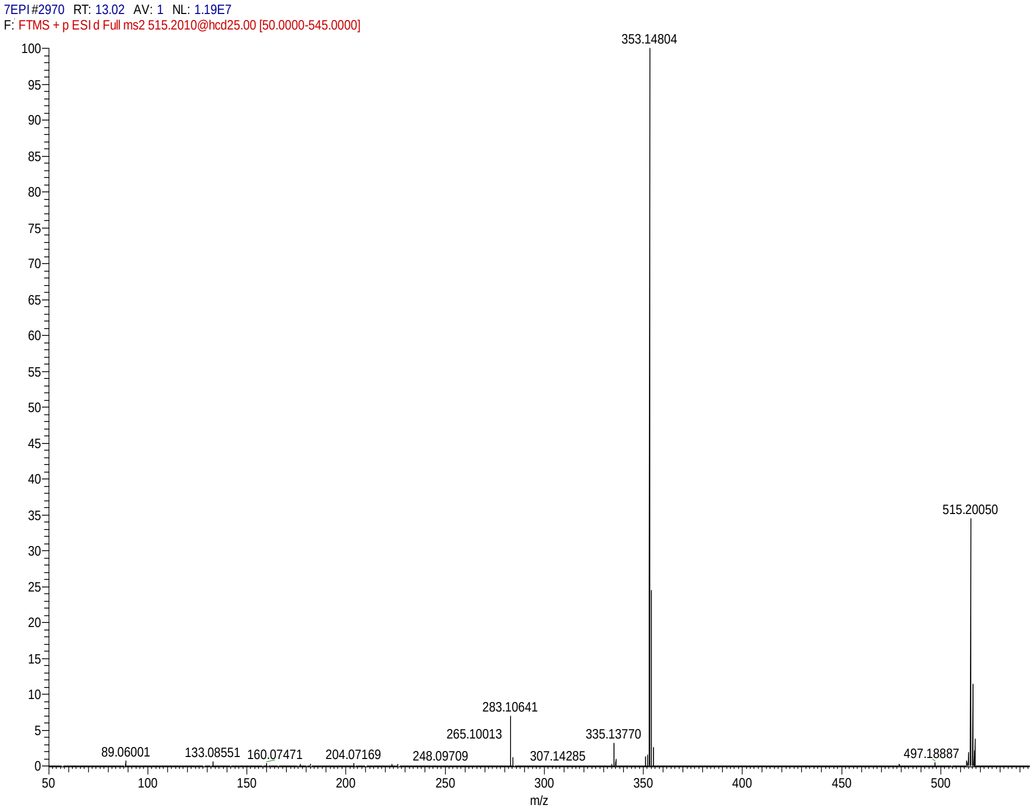

Figure S11 The HR-ESI-MS of compound **1**

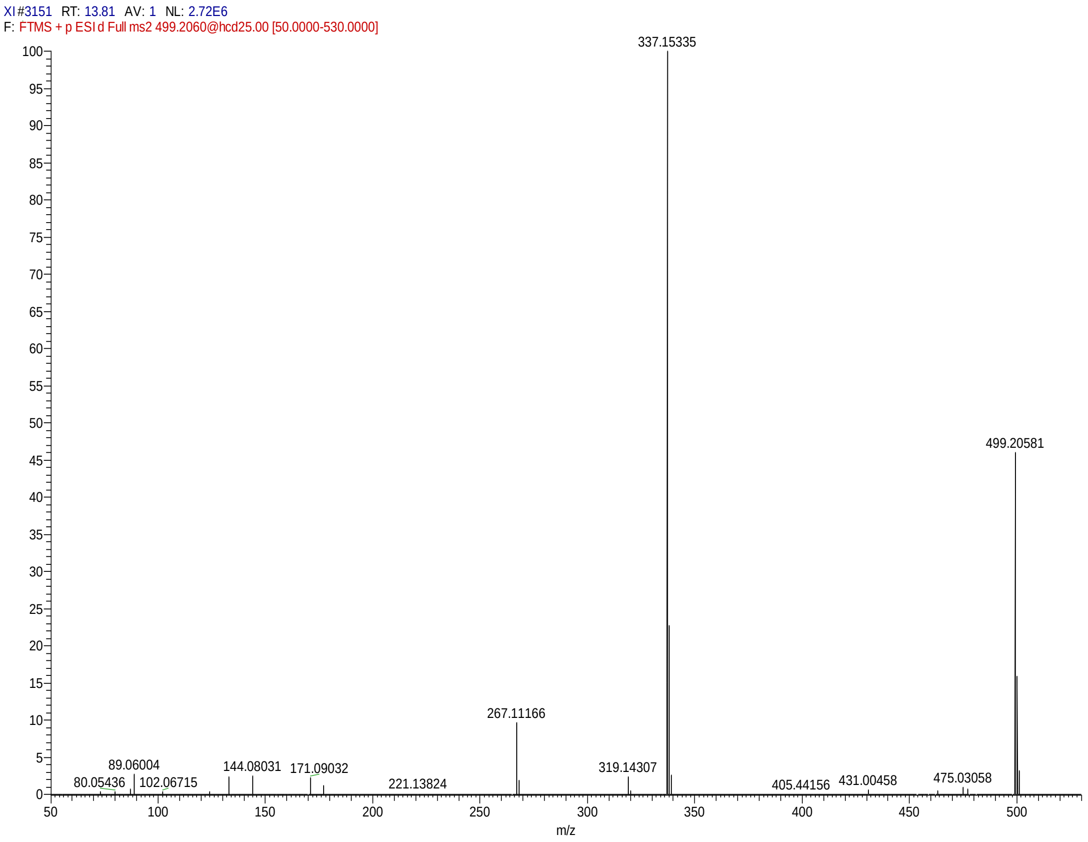

Figure S12 The HR-ESI-MS of compound **2**

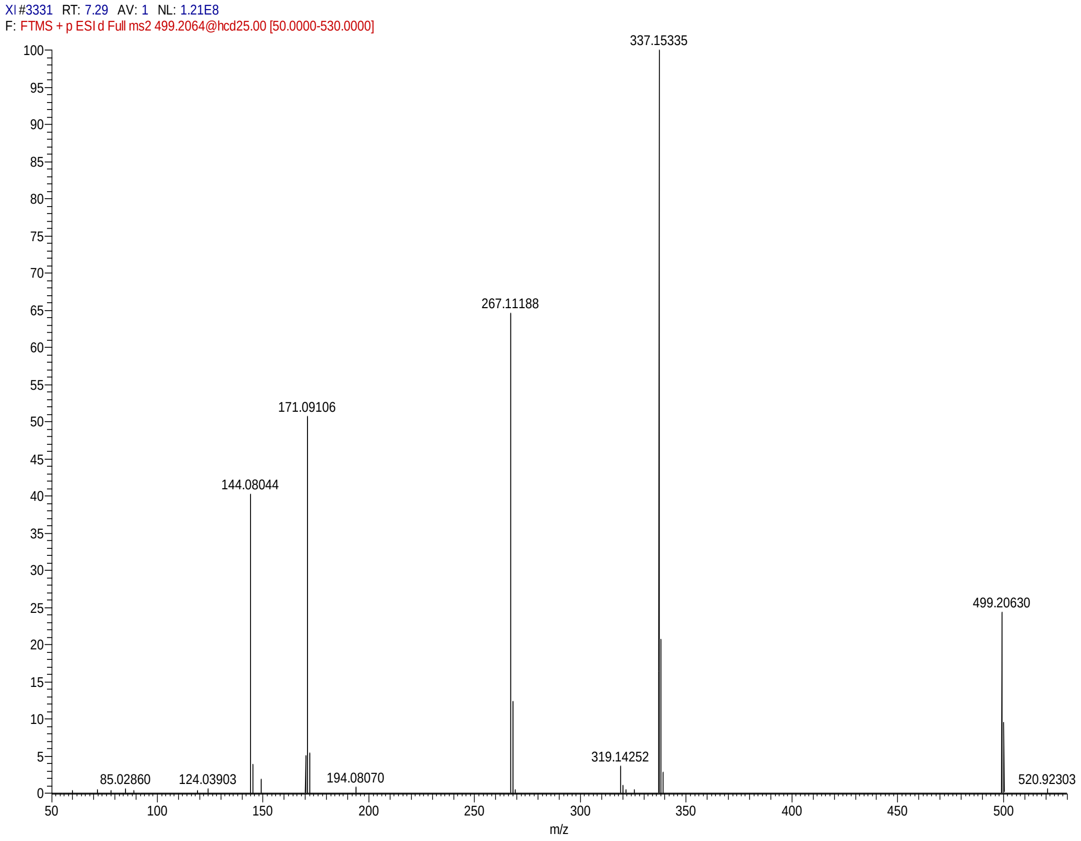

Figure S13 The HR-ESI-MS of compound **3**

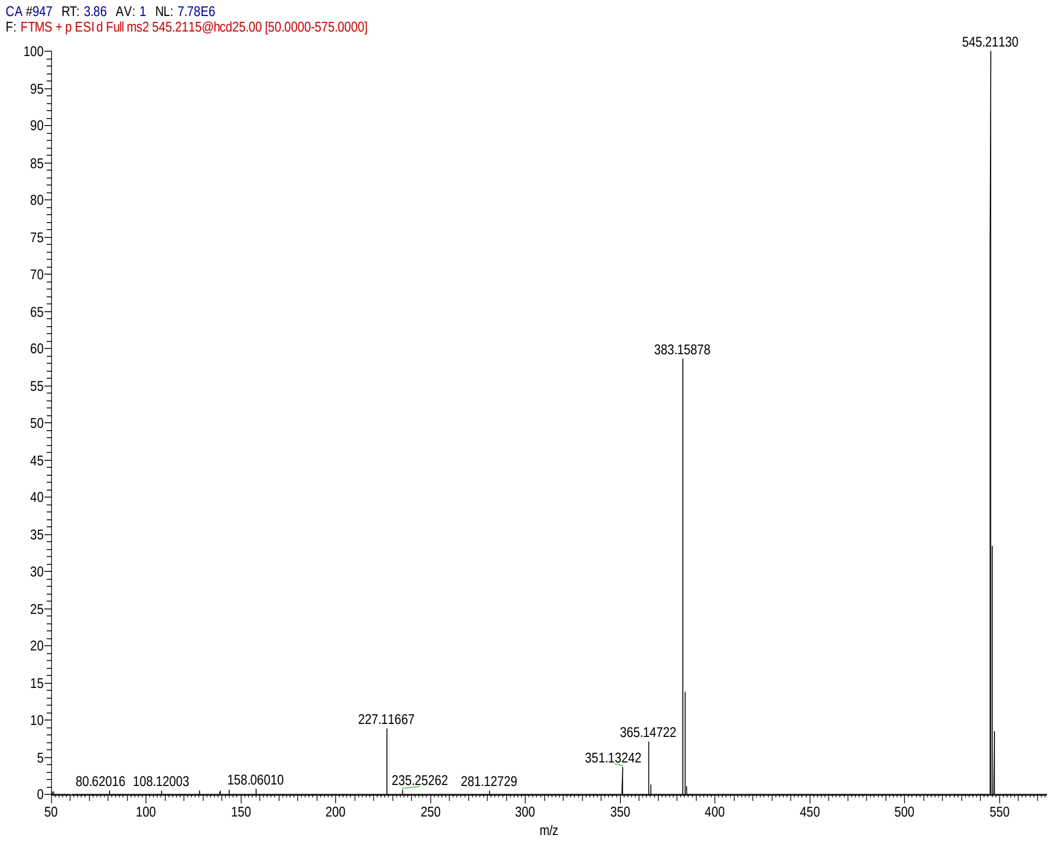

Figure S14 The HR-ESI-MS of compound **4**

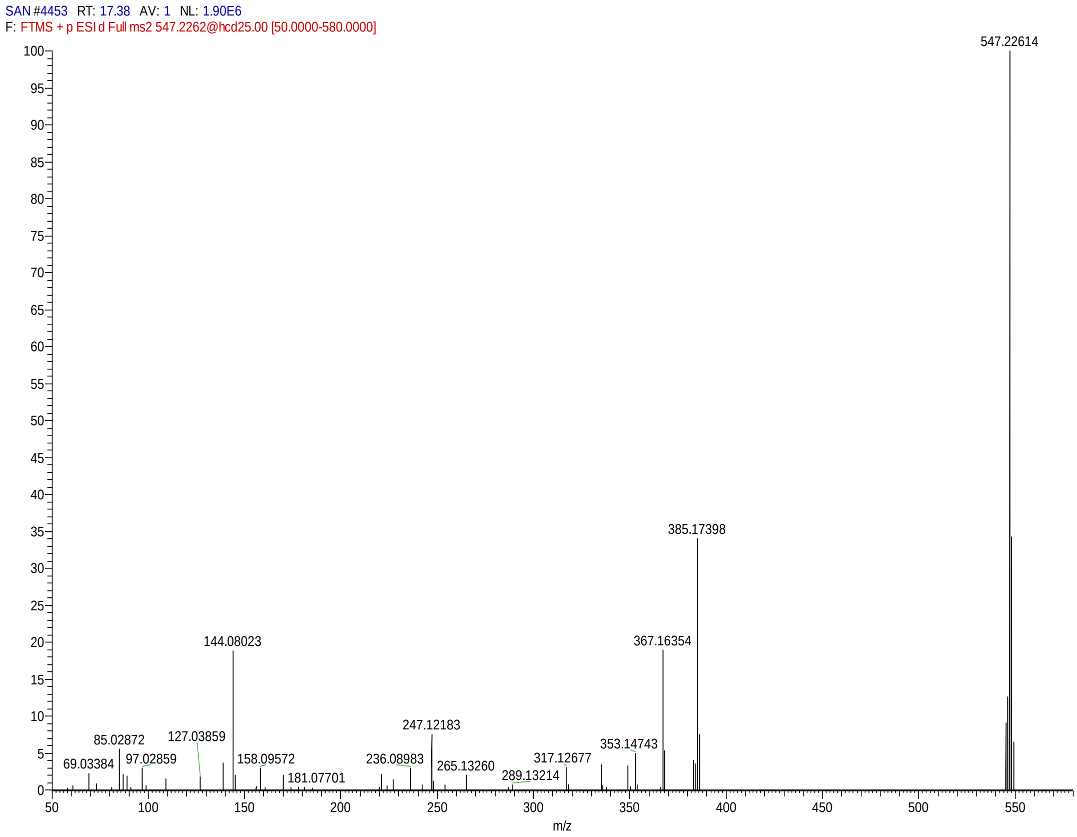

Figure S15 The HR-ESI-MS of compound **5**
